# Non-invasive assessment of skin hydration and sensation with diffuse reflectance spectroscopy

**DOI:** 10.1038/s41598-023-47349-5

**Published:** 2023-11-17

**Authors:** Ying-Yu Chen, Shih-Yu Tzeng, Yun-Yo Yen, Nan-Yu Cheng, Sheng-Hao Tseng

**Affiliations:** 1 Department of Photonics, National Cheng-Kung University, Tainan, 701 Taiwan, ROC; 2 Research Development and Innovation Center, Show Chwan Health Care System, Changhua City, 500 Taiwan, ROC; 3 Department of Health-Business Administration, Fooyin University, Kaohsiung, 831 Taiwan, ROC; 4 School of Dentistry, College of Dental Medicine, Kaohsiung Medical University, Kaohsiung, 807 Taiwan, ROC

**Keywords:** Biomedical engineering, Optics and photonics, Applied optics

## Abstract

The skin is a vital organ in the human body, providing essential functions such as protection, sensation, and metabolism. Skin hydration is one of the crucial factors in maintaining normal skin function. Insufficient skin hydration can lead to dryness, shedding of the stratum corneum, a decrease in skin barrier function, and may cause skin inflammation. Therefore, maintaining or improving skin hydration is critical in promoting healthy skin. Currently, the commonly used method for measuring skin hydration is bioelectrical capacitance analysis, which is often affected by environmental humidity and can only provide limited information. To overcome these limitations, this study used diffuse reflectance spectroscopy (DRS) in the wavelength range of 400-1000 nm to quantify skin absorption and scattering modulation caused by changes in skin hydration states. The advantages of this technique include rapid measurements, non-invasiveness, a straightforward optical setup, and suitability for prolonged skin monitoring. We found that DRS-derived skin absorption coefficients had a correlation coefficient of 0.93 with the skin capacitance at various skin hydration states. In addition, our findings reveal that absorption and scattering coefficients may be useful in discerning skin hydration enhancement induced by applying soaked cotton pads or cosmeceutical facial masks, as well as evaluating skin sensation. This study verifies that the DRS method could be a convenient and effective tool for evaluating skin hydration related information.

## Introduction

Skin is a protective layer covering the body and an organ for various defense and regulating functions, including retaining water and regulating body temperature. Dermatologists and scientists have used skin biopsies to investigate skin properties^1–4^. In recent decades, noninvasive methods and systems have been developed to monitor the variation of skin properties, for example, skin hydration^5,6^, trans-epidermal water loss (TEWL)^7^, sebum volume^8^, skin pH^9^, and so on. The evaluation of skin hydration level has attracted great attention in the medical field as it helps to understand the characteristics of an individual's skin and diagnose skin diseases^10^. For instance, Zainal et al. demonstrated that skin hydration could be determined by measuring skin capacitance and could be used to quantify the degree of recovery in atopic dermatitis treatment^11^. In general, several methods are available currently to quantify skin hydration states, such as bioelectrical capacitance or impedance measurements^5,12,13^, confocal Raman microscopy (CRM)^6,14,15^, nuclear magnetic resonance spectroscopy (NMRS)^16,17^, Fourier transform near-infrared spectroscopy (FTIRS), and near-infrared spectroscopy^18^. Among all the listed methods, measuring skin electrical properties has been the most widely adopted method for quantifying skin hydration levels due to their relatively low cost and compact footprint^10^. By measuring the electrophysiological parameters (conductivity, electrical impedance, capacitance) of the skin, one could indirectly evaluate the water content of the skin^5,12^. However, there are reports indicating that the electrical conductance methods would be affected by blood and exocrine sweat gland function^13^. The CRM method couples a Raman spectrometer to a standard optical microscope, allowing for highly magnified visualization of a sample and Raman analysis with a microscopic laser spot. By analyzing the scattered light, the molecular composition, structure and relative content can be learned. In addition, the spatially resolved molecular structure information inherent in the Raman spectra yields insight into penetration and hydration mechanisms^6,19^. In 2019, Maxim and colleagues successfully determined the water mass percentage in the high wavenumber region by calculating the ratio of the area under the curve of the OH Raman band (3350–3550 cm^−1^) to the CH_3_ vibration band of keratin (2910–2965 cm^−1^)^14^. However, CRM systems usually have a considerable footprint, and the single-point scanning mechanism would take a long time to achieve a 1-D or 2-D scan and thus is not suitable for the exploration of the skin hydration dynamic changes^14^. NMRS measures the resonance of hydrogen, basically the protons of the water molecules, under the effect of the magnetic field. While NMRS is bulky and takes a long time to scan, it is one of the direct hydration measurement methods for the evaluation of the overall moisture content in the epidermis and outer dermis^16^. FTIRS methods are broadly available in monitoring water content and often used for process and quality control in industrial applications^20^. The sample irradiated with infrared light absorbs the light, and the matching molecular vibrations are activated. Spectral frequencies were analyzed by the Fourier transform, and varying molecular groups (predominantly N–H, C–H, and O–H bonds) at typical wavelengths were distinguished with absorption bands. Therefore, some research teams used FTIRS to determine the water content in the skin^21,22^. However, in addition to the high noise problem, FTIRS is unable to monitor skin properties over extended periods^23^.

Diffuse reflectance spectroscopy (DRS) in 800–2000 nm wavelengths has recently been used to determine water content of in-vivo skin^24–26^. On account of the fact that NIR(near-infrared) spectroscopy is a nondestructive, and noninvasive method, and water has strong NIR absorptions centered near 970 (second overtone of water absorption) and 1450 nm (first overtone of water absorption), evaluation of water content in the skin can be quantified by analyzing the characteristics of the skin spectrum^25^. The advantage of DRS is that it does not require a complex optical setup, and the measurement time is short allowing one to conduct skin short-term change studies. However, the disadvantage of DRS is that it possesses a very weak z resolution, and the measurement result represents the average property of a certain skin volume depending on the separation of the light source and detector as well as the individual's skin optical properties. By using appropriate algorithms, DRS allows identifying the absorption and scattering characteristics of biological tissue, and thus various tissue contents such as hemoglobin and water concentrations can be deduced^27–32^. Traditionally, photon diffusion theory is used for deep tissue studies where reflectance measurement setup is configured with large source detector separations (SDS), such as in breast cancer research^33^, and brain research ^34^. It has rarely been used for setups with short source-detector separations for superficial tissue investigation due to the breakdown of the diffusion approximation in the derivation of the photon diffusion model^35^. In the past decade, we have demonstrated that the photon diffusion model can precisely translate the diffuse reflectance spectra to the absorption and reduced scattering spectra of superficial tissues by using an optical fiber probe equipped with a high scattering layer to diffuse light efficiently at short source detector separations^29,36,37^. This unique surface-diffusion design involves coupling the source fiber to a Spectralon slab, characterized by its high scattering and low absorption properties. As light is emitted from the fiber, it undergoes multiple scattering events within the Spectralon slab before penetrating the target tissue for measurement. The detection fiber interfaces directly with the sample but within the confines of the slab. A significant aspect of our probe is that its detection depth is predominantly influenced by the source-detector separation and the thickness of the scattering layer. This design allows our probe to focus measurements primarily on the skin's superficial layers, enabling accurate quantification of skin components such as collagen, bilirubin, hydration, and hemoglobin^32,36–38^. Based on our laboratory's research^39^, this specialized probe has an interrogation depth of up to 300 μm in Asian skin, encompassing the stratum corneum, epidermis, and reaching the upper dermis.

The purpose of this study is to determine the skin absorption and scattering modulation induced by the variation of skin hydration state using diffuse reflectance spectroscopy. In this study, we induced changes in the skin hydration of the subjects by using soaked cotton pads and cosmeceutical facial masks. The measurement results were compared to those derived from the bioelectrical capacitance method. Finally, we will discuss the implications of our findings in facilitating the understanding of skin property variation dynamics during changing hydration states.

## Results

### DRS measurement results for two skin treatment protocols

**Figure 1:**
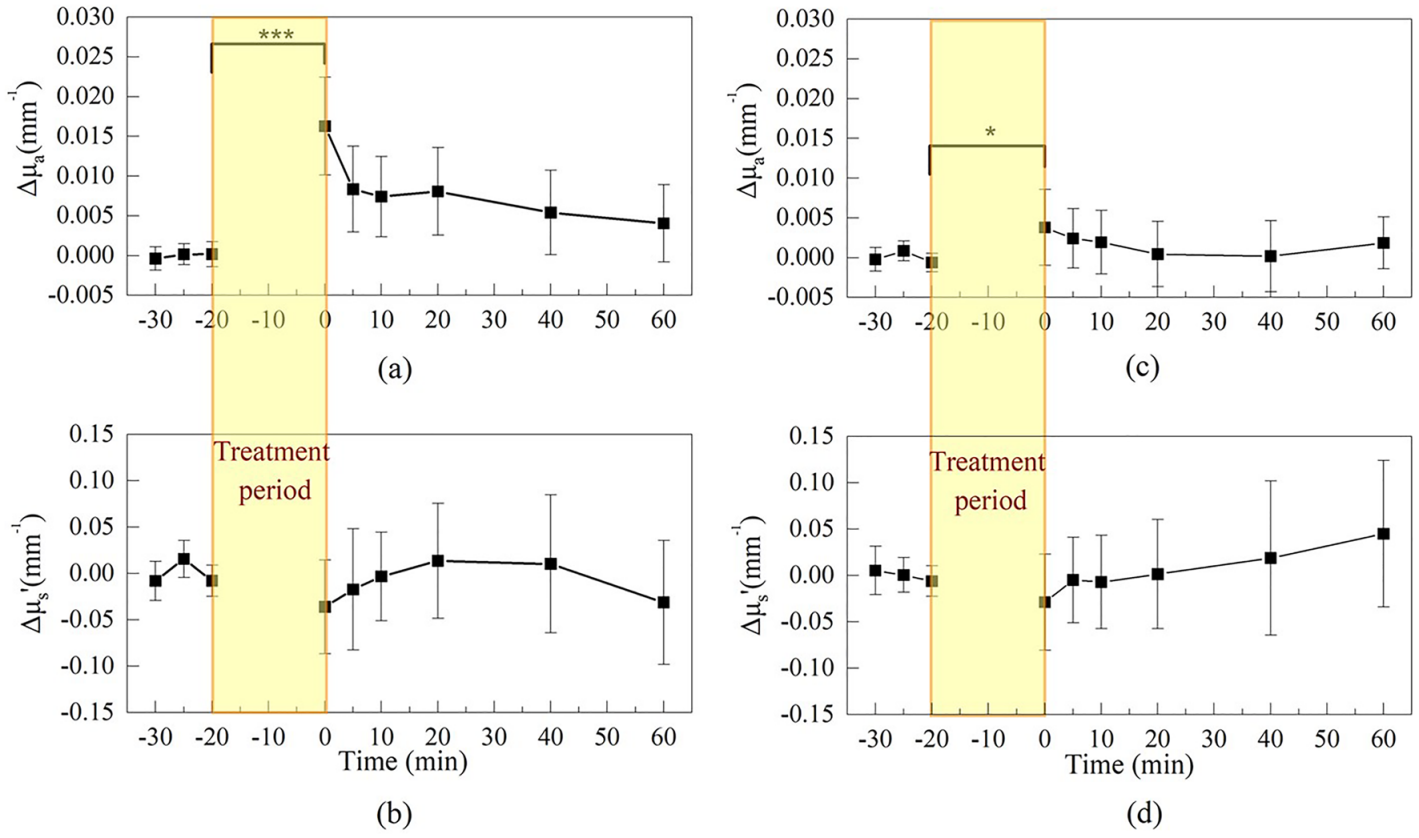
(**a**) Changes of absorption coefficient and (**b**) reduced scattering coefficient in facial mask experiment. (**c**) Changes of absorption coefficient and (**d**) reduced scattering coefficient in soaked cotton pad experiment. The initial data point after hydration treatment is considered as the 0-min time point. *P < 0.05 and ***P < 0.001. (Removal of facial mask or soaked cotton pad was taken as the 0-min time point.)

We conducted an analysis of the changes in absorption coefficient and reduced scattering coefficient at 980 nm for the two skin treatment protocols. Figure 1 illustrates the average changes in skin optical properties at 980 nm of 10 subjects before and after using a moisturizing facial mask and a soaked cotton pad, respectively. We calculated the baseline by averaging the first three absorption coefficient measurements. The value of ∆µ_a_ was obtained by subtracting each data point from the baseline value, while ∆µ_s_' was calculated in the same manner as ∆µ_a_. After the facial mask was removed (Fig. 1a), the absorption coefficient showed a mean increase of 0.016 ± 0.006 (1/mm), estimated increase in SC hydration of 20% derived from GPSkin Barrier®, and the Student’s t-test demonstrated a significant difference (P = 5.45E−5, n = 10) between the time points of − 20 min and 0 min. There was no significant change (P = 0.11, n = 10) observed in the reduced scattering coefficient (Fig. 1b). On the other hand, in the soaked cotton pad application experiment, the average absorption coefficient increased by 0.0038 ± 0.005 (1/mm), estimated increase in SC hydration of 10% derived from GPSkin Barrier®, as can be seen in Fig. 1c, which was also statistically different from that before application (P = 0.04 between − 20 min and 0 min point, n = 10). Similar to the facial mask experiment, there was no significant difference in reduced scattering coefficient before and after cotton pad application. (*P* = 0.27, n = 10, Fig. 1d).

### Relative electrical conductivity and TEWL values for the two skin treatment protocols

**Figure 2:**
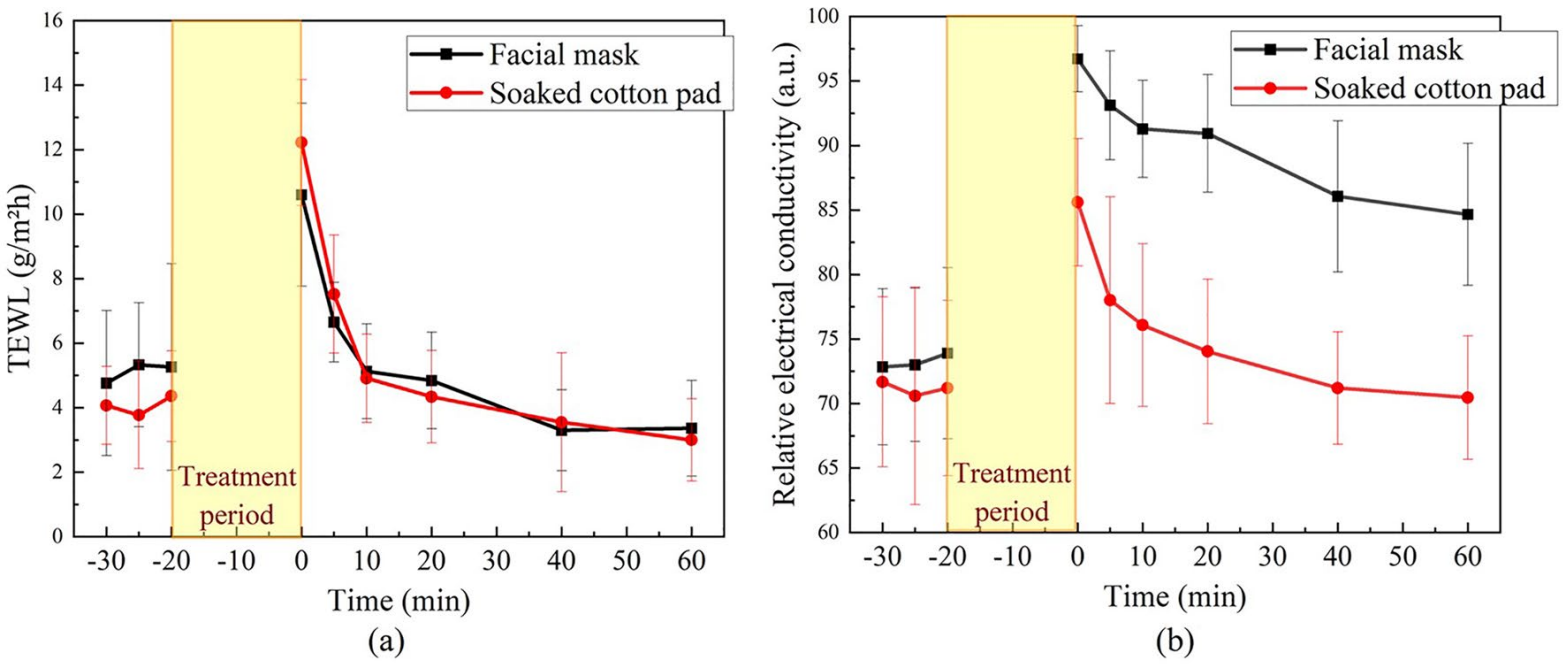
(**a**) TEWL change chart and (**b**) relative electrical conductivity change chart of the facial mask (black square) and soaked cotton pad (red dot) experiments. (Removal of facial mask or soaked cotton pad was taken as the 0-min time point.)

In this study, we utilized TEWL and relative electrical conductivity as a reference to assess the ability of DRS in measuring skin water content variation. Figure 2 shows the alterations in TEWL and relative electrical conductivity under two skin treatment methods. From Fig. 2b, it can be seen that the increase in skin electrical conductivity induced by the use of a facial mask is greater than that induced by soaked cotton pads. However, there is no significant difference in the TEWL values of these two treatments as shown in Fig. 2a. Specifically, in the baseline of the facial mask experiment, the average TEWL of 10 subjects was around 5.0 g/m^2^h, and the relative electrical conductivity was 73.9 (a.u.) on average. After the treatment, the average relative electrical conductivity increased to 96.7 (a.u.), and the average TEWL correspondingly increased to 10.6 g/m^2^h (112%). On the other hand, in the baseline of the soaked cotton pad experiment, the average TEWL was around 4.0 g/m^2^h, with an average relative electrical conductivity of 71.2 (a.u.). After the treatment, the average relative electrical conductivity increased to 85.6 (a.u.), and the average TEWL correspondingly increased to 12.2 g/m^2^h (205%). In the subsequent discussion, further statistical analyses will be conducted on the optical parameters, as well as the TEWL and relative electrical conductivity.

### Cutaneous sensation evaluation results of subjects under two skin treatment protocols

**Figure 3:**
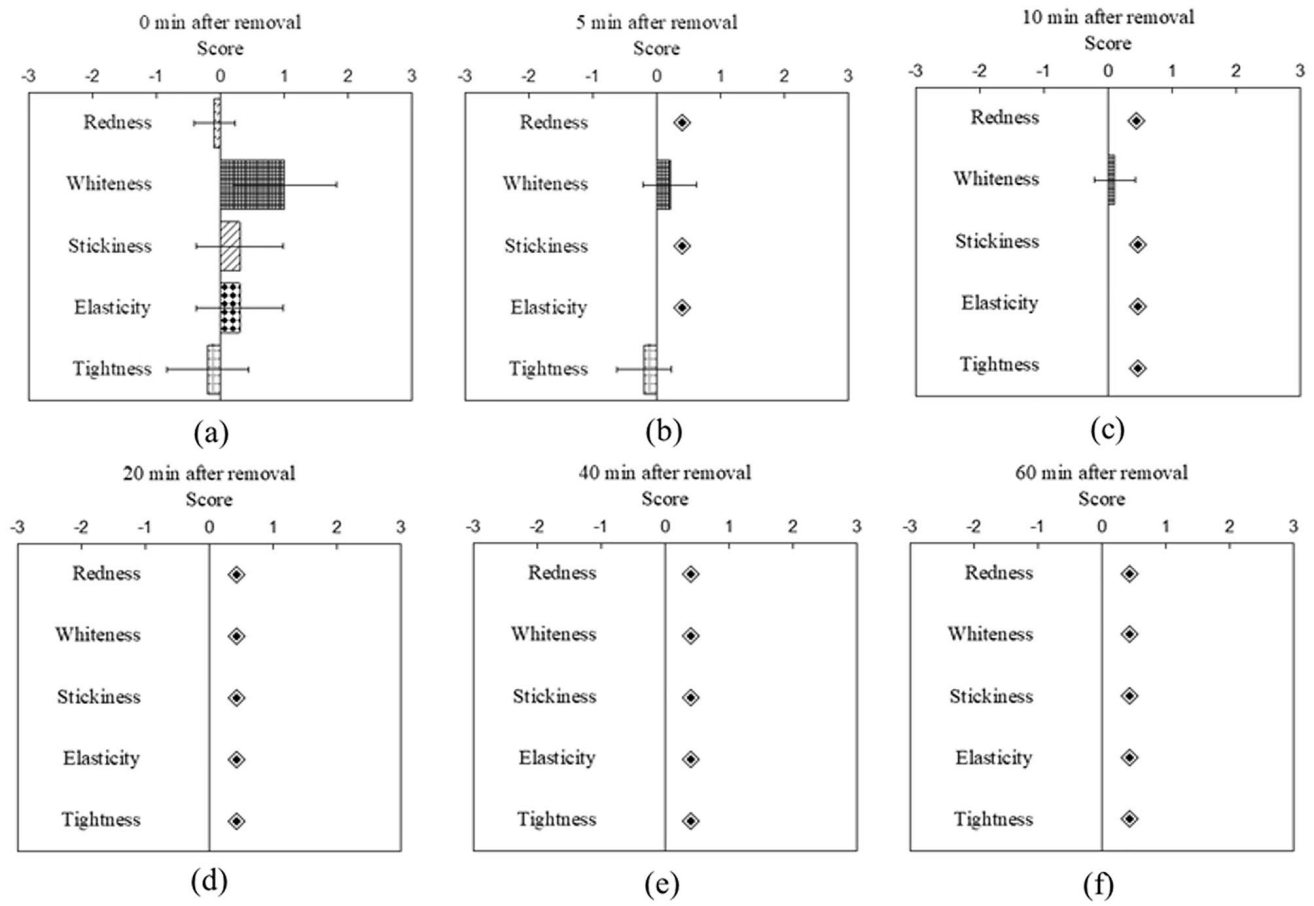
The participants' ratings of cutaneous sensation, including redness, whiteness, stickiness, elasticity, and tightness, were evaluated at 0 (**a**), 5 (**b**), 10 (**c**), 20 (**d**), 40 (**e**), and 60 (**f**) mins after the removal of the facial mask. (◈ No feelings).

**Figure 4:**
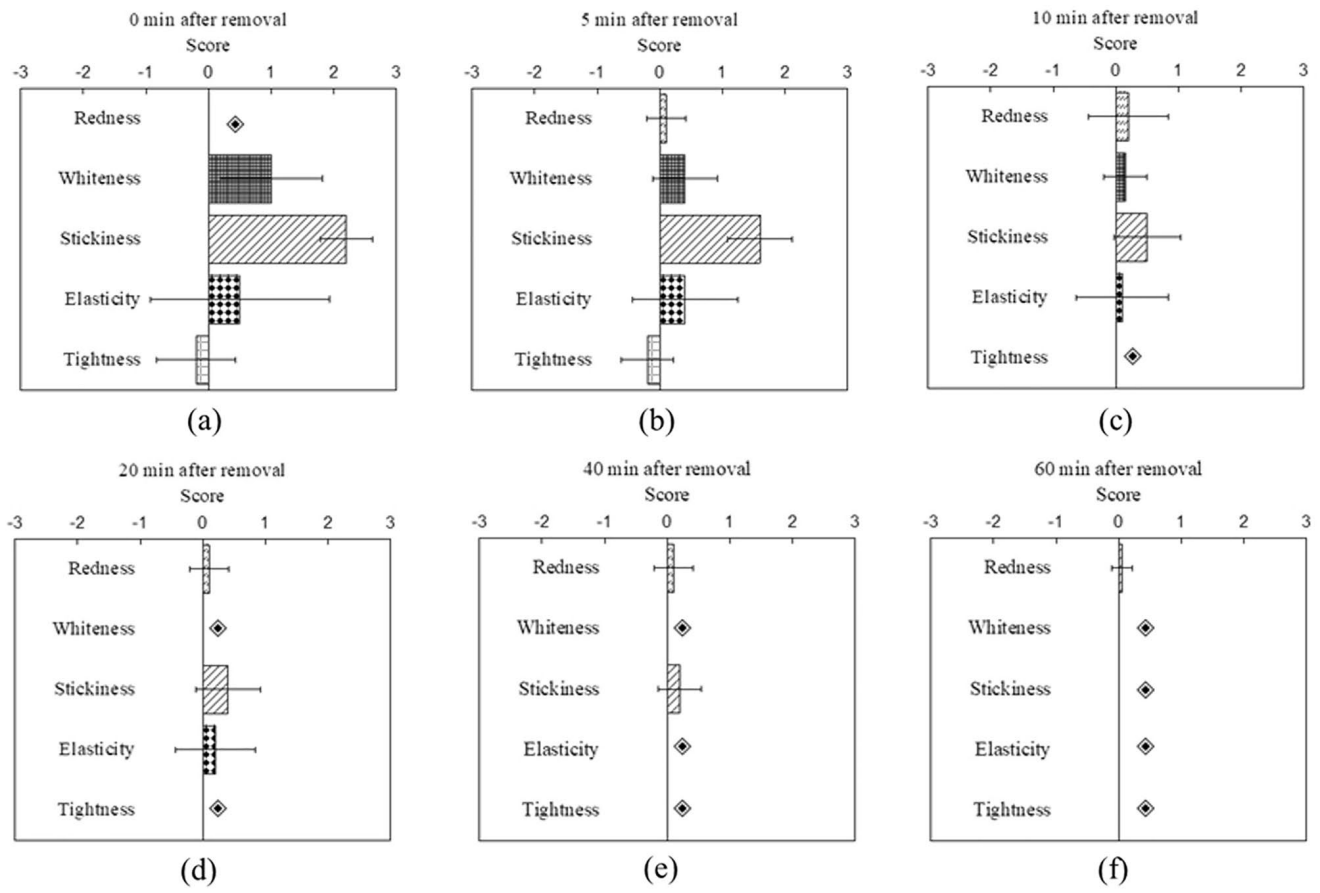
The participants' ratings of cutaneous sensation, including redness, whiteness, stickiness, elasticity, and tightness, were evaluated at 0 (**a**), 5 (**b**), 10 (**c**), 20 (**d**), 40 (**e**), and 60 (**f**) mins after the removal of the soaked cotton pad. (◈ No feelings).

After the removal of the facial mask or soaked cotton pad, participants were asked to fill out a cutaneous sensation questionnaire to evaluate the skin redness, whiteness, stickiness, elasticity, and tightness at 0, 5, 10, 20, 40, and 60 min. Figures 3 and 4 present the average ratings of all participants’ cutaneous sensation at different time points after a 20-min application of a facial mask and soaked cotton pad, respectively. In the facial mask group, the measurement sites of all test subjects appeared whitened, while some of them had a reddish appearance (Fig. 3). On average, the sensory evaluations of redness, whiteness, elasticity, and stickiness showed a strong initial perception that gradually decreased over time. Conversely, with regard to tightness, the cutaneous sensation was classified into two categories, as some individuals experienced tightness while others perceived an increase in skin softness.

Additionally, it was found that cutaneous sensations of elasticity and tightness were relatively consistent, as those who perceived an increase in skin elasticity also experienced an increase in tightness. All cutaneous sensations mentioned above dissipated after 60 min of removing the facial mask. On the other hand, in the soaked cotton pad experiment, whiteness, stickiness, and elasticity all increased on average after removing the soaked cotton pad and decreased over time. Particularly, after applying the soaked cotton pad, some individuals experienced a reduction in redness, resulting in a negative score for redness on average, which differed from the facial mask experiment. The sensations of stickiness and elasticity were only present immediately after the removal of the soaked cotton pad at 0 min and subsided quickly thereafter. Last but not least, 7 subjects rated a zero tightness score after applying water, while 3 subjects rated negative tightness scores (softer skin).

Our results revealed that, compared to the baseline ratings, both skin treatment protocols resulted in higher average ratings of cutaneous sensation for whiteness, stickiness, and elasticity, while the rating for tightness had great variation among subjects. Notably, the sensation of applying a facial mask was more prominent and longer-lasting than that of a soaked cotton pad. After 10 min of removing the soaked cotton pad, most scores returned to the baseline level, while stickiness and elasticity remained high even after 20 min of removing the facial mask. This phenomenon will be discussed further in later paragraphs.

## Discussion

**Figure 5:**
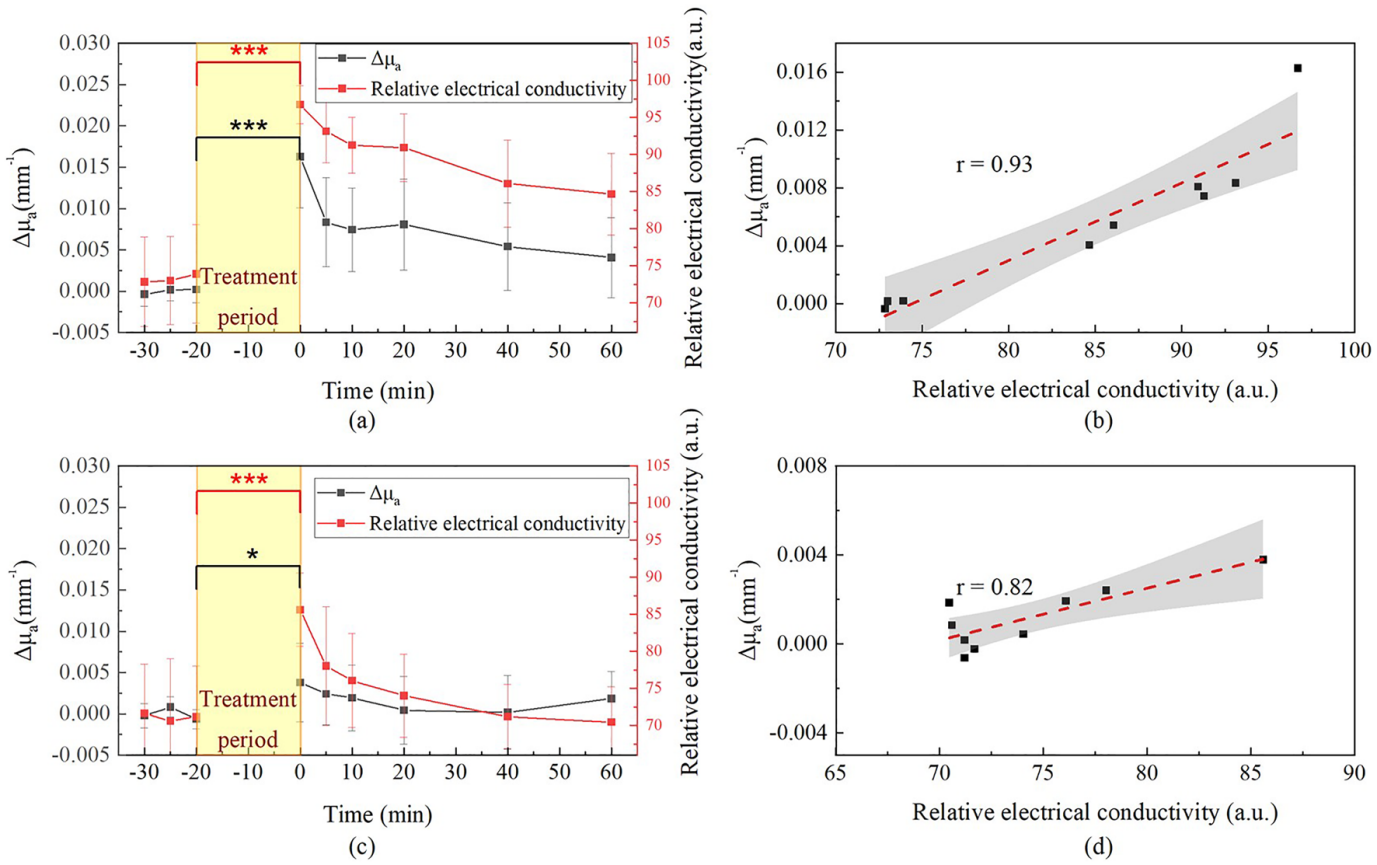
(**a**) Absorption coefficient differences and relative electrical conductivity values of the facial mask experiment and (**b**), their correlation plot. (**c**) Absorption coefficient differences and relative electrical conductivity values of the soaked cotton pad experiment and (**d**), their correlation plot. *P < 0.05 and ***P < 0.001. (Removal of facial mask or soaked cotton pad was taken as the 0-min time point).

It has been reported in the literature that changes in skin absorption coefficients can be caused by variations in skin hydration, which is due to the contribution of the optical absorption of water^40^. However, there is no literature comparing whether there are differences in absorption and scattering coefficients between different skin hydration treatment protocols. Therefore, we designed two skin hydration treatment protocols in this study. The 980 nm wavelength is recognized as the characteristic wavelength for water absorption in the spectrum^41^. In order to understand the relationship between the absorption coefficients at 980 nm measured by our DRS system and the skin’s relative electrical conductivity, we performed a correlation analysis between them. We extracted data from Fig. 1a and 2b and re-plotted them in Fig. 5a and c for the facial mask and soaked cotton pad experiments, respectively.

It can be observed in Fig. 5a and c that the application of a facial mask and soaked cotton pad to the skin resulted in an increase in skin’s relative electrical conductivity. Specifically, the skin’s relative electrical conductivity increased by around 25% from the baseline (P = 9.66E−7) with the use of a facial mask, as shown in Fig. 5a, and increased by approximately 15% from the baseline (P = 1.97E−6) with the use of a soaked cotton pad, as shown in Fig. 5c. As discussed in Results. A, the skin absorption coefficient at 980 nm significantly increased (P < 0.05) after removal of the soaked cotton pad and facial mask. Moreover, the increase in absorption at 980 nm was greater in the facial mask treatment than in the soaked cotton pad treatment, with an approximate difference of 0.0122 (1/mm). In addition, it was found that the correlation between the relative electrical conductivity values and absorption coefficient variation was r = 0.93 in the facial mask experiment (Fig. 5b), and r = 0.82 in the soaked cotton pad experiment (Fig. 5d). These results suggest that there is a high degree of correlation between the absorption coefficient variation and relative electrical conductivity, indicating that 980 nm absorption can serve as a reliable measure for quantifying skin moisture.

From the perspective of the absorption coefficient and relative conductivity, notable differences were observed following the facial mask treatment. As discussed in the literature^42^, the traditional function of moisturizers is to replenish the intercellular lipid layer and natural moisturizing factors, as well as to form a lipid film on the skin surface to reduce transdermal water loss and improve hydration. We postulate that this difference may be attributed to the nature moisturizing factor (NMF) present in the facial mask. The stratum corneum ascribes most of its flexibility and water-holding properties to hygroscopic, water-soluble compounds, dubbed NMF^43–45^. NMF is stored in SC cells in highly concentrated forms, taking up 20% to 30% of SC’s dry weight^46^. Through absorbing moisture in the atmosphere and dissolving it in their own water of hydration, hygroscopic NMF can serve as a very effective moisturizer^47^. In the past, researchers such as Maxim Morin discovered that by applying this NMF formula to multiple areas of the skin, it significantly increased the moisture accumulation rate (MAT) of the applied area^45^. Facial masks used in this study contain NMFs, such as vitamin B3, PCA-NA moisturizing factors and amino acid. These factors increase the moisture accumulation rate in the skin and boost hydration in SC. Therefore, the observed increase in skin hydration after the facial mask treatment in this study could be attributed to the influence of NMF within the stratum corneum. NMF enhances hydration levels and fortifies the skin barrier, ultimately impeding the evaporation of moisture from the skin. This finding is consistent with prior research in the field^48^.

Building on the results presented in section C, we will discuss the implications of cutaneous sensation and its relationship with optical parameters. Whiteness may be associated with surface reflection, while redness may be related to individual skin microcirculation enhancement. Stickiness may be attributed to the NMF of facial mask components, while tightness and elasticity may be associated with cellular hydration and swelling^49^. We observed that, during hydration experiments, sensations of whiteness, redness, and stickiness gradually diminished after the removal of the facial mask and soaked cotton pad. Throughout the experiments, we observed that out of the 10 participants, 7 felt their skin become tauter after facial mask removal, while the remaining 3 felt their skin become softer. Therefore, those who experienced tightness also perceived their skin to be more elastic after application, whereas the other 3 participants did not report any noticeable sensation.

**Figure 6:**
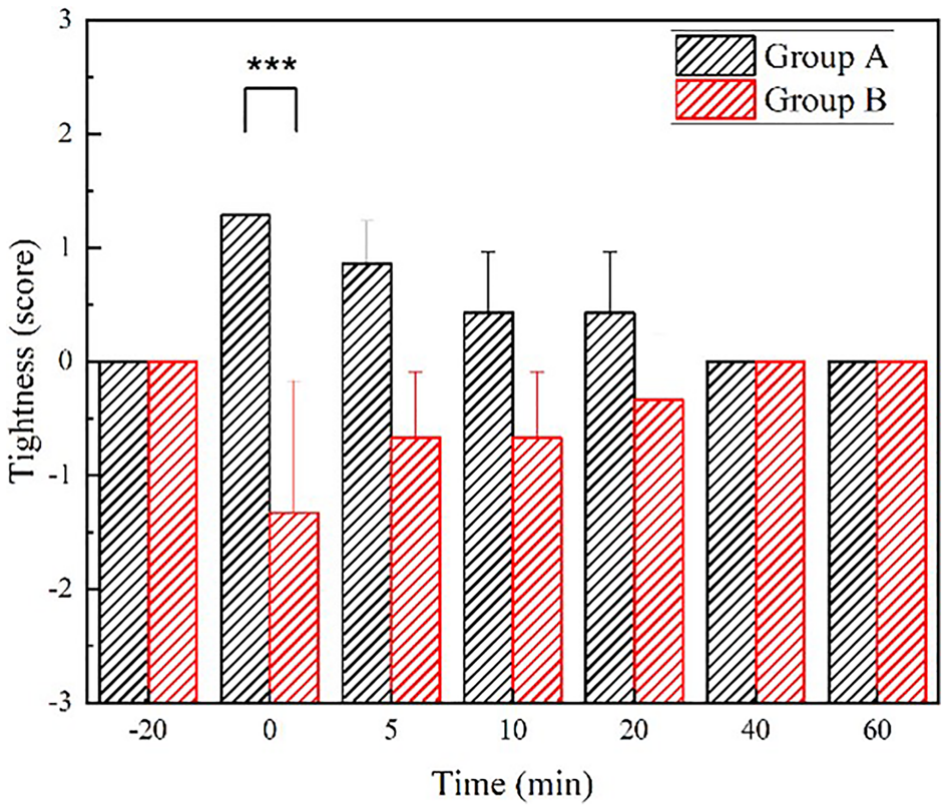
Tightness scores time progression of Group A, comprising 7 individuals that reported a more significant degree of tightness, and Group B, consisting of 3 individuals that reported experiencing less tightness. ***P < 0.001. (Removal of facial mask was taken as the 0-min time point.).

We suspected that this skin tightness sensation could be a discerning feature for separating the 10 participants. To this end, we further divided the test subjects into two groups based on their cutaneous sensation in tightness as shown in Fig. 6. In Group A, participants perceived a greater degree of tightness at the measurement site (7 individuals), while in Group B (3 individuals), a soft sensation was reported in the facial mask experiment. Groups A and B had significantly different tightness scores right after facial mask removal, and their differences subsided over time. Previous research had utilized the use of immunohistochemical procedures numerous nerve fibers have been found in all cell layers of human epidermis. Almost all nerve fibers basal branches divide distally in the epidermis and eventually end in small enlargements, usually in the stratum granulosum^61^. Thus, we speculate that the 7 people that experienced more tightness (Group A), the moisture diffused into the stratum granulosum, the sensory neurons in the stratum granulosum sensed the cells swelling, therefore giving out the sense of tightness. As for the other three people (Group B), the moisture might only stay in the SC, and didn’t seep into the stratum granulosum; as a result, the neurons were not stimulated. This inference is consistent with previous studies that have investigated the 'liquid–liquid' interface in the skin. In addition to the air–liquid interface barrier in the SC, the tight-junction strand (TJs) is also important as a ‘liquid–liquid’ interface barrier’ in the epidermis. They are responsible for sealing epithelial cells to form a functional barrier of the paracellular pathway^44^. It was experimentally demonstrated that the stratum granulosum (SG) second cell layer is sealed together by functional TJs in the epidermis^50,51^. Based on this information, under the effect of exterior changes in skin hydration, the most depth that this could affect is to the first layer of the stratum granulosum, any depth more, it would be shielded by the ’liquid–liquid’ interface barrier of TJs. The effect of water immersion on skin properties has been widely discussed in several studies^49,52^. The ability of the epidermis to take up water^53^ results in a thickening and swelling. Water is accumulated in intracellular spaces, and corneocytes swell^54^. Furthermore, water disrupts the intercellular lipid structures and leads to a three to four-fold increase in the stratum corneum thickness^49^ and the skin becomes tighter.

**Figure 7:**
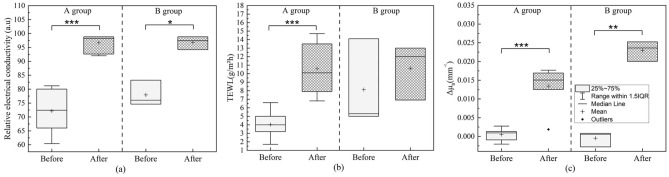
Boxplots of (**a**) relative electrical conductivity, (**b**) TEWL, and (**c**) differences in absorption coefficient before and after the removal of the facial mask for Group A and Group B. *P < 0.05, **P < 0.01 and ***P < 0.001.

Figure 7a–c illustrate the boxplots of relative electrical conductivity, TEWL, and differences in absorption coefficient before and after the removal of the facial mask for Group A and Group B, respectively. Group A exhibited significant differences in the changes of the conductivity coefficient (Fig. 7a, P = 2.18E−5), TEWL (Fig. 7b, P = 8.53E−4), and absorption coefficient Fig. 7c, P = 8.48E−4), before and after facial mask treatment. On the other hand, Group B exhibited significant differences in the changes of the conductivity coefficient (Fig. 7a, P = 0.04) and absorption coefficient (Fig. 7c, P = 0.01) before and after facial mask treatment, while no significant difference was observed in TEWL (Fig. 7b, P = 0.66). It can be observed from Fig. 7a and c that the changes in absorption coefficient and relative electrical conductivity are closely related parameters.

**Figure 8:**
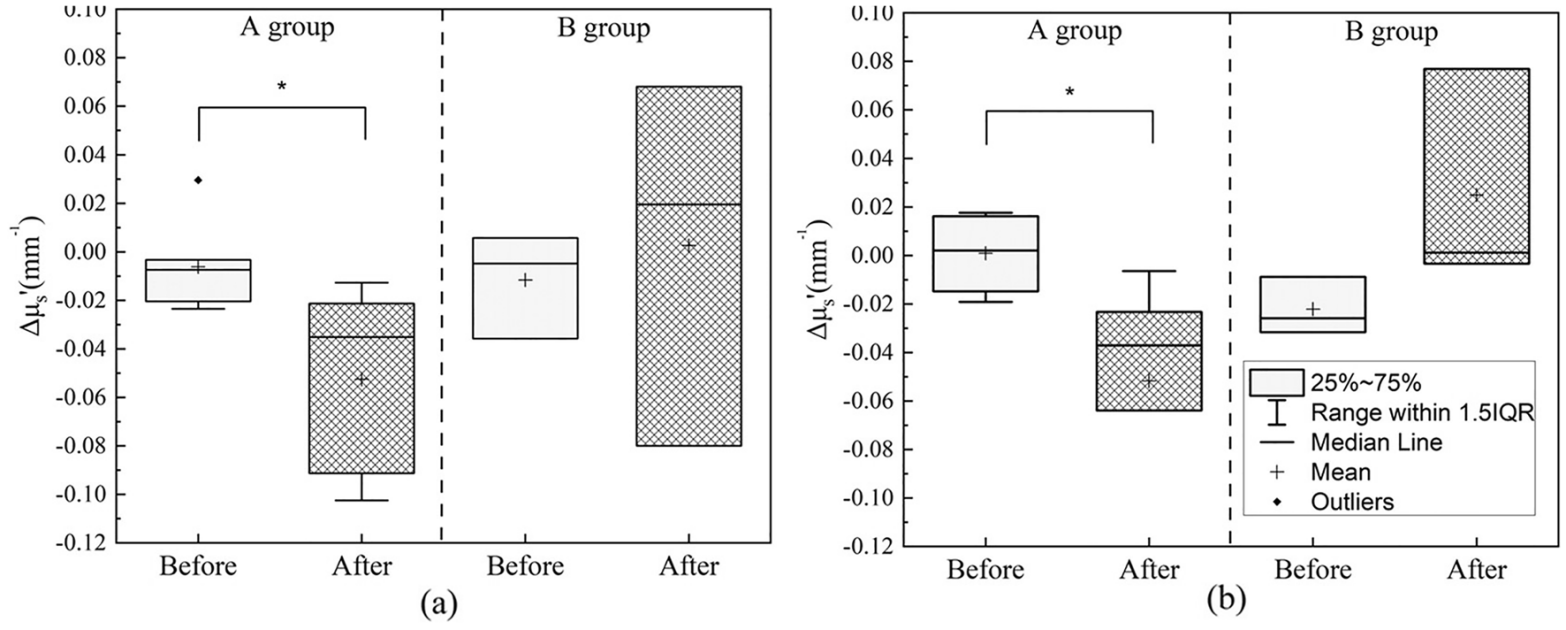
Boxplots of differences in reduced scattering coefficient before and after the removal of the (**a**) facial mask and (**b**) soaked cotton pad for Group A and Group B. *P < 0.05.

As shown in Fig. 1, there was no significant difference in the mean scattering coefficient of ten participants before and after the two skin hydration treatment protocols. However, if the subjects are divided into Groups A and B, significant differences in the scattering coefficients before and after skin hydration treatments can be observed. Figure 8 shows boxplots of the changes in scattering coefficients before and after the two treatment protocols. As demonstrated in Fig. 8a, it was observed that the scattering coefficient of Group A’ averages lower after facial mask (0.053/mm, P = 0.02), whereas Group B, that experienced less tightness, did not exhibit a significant alteration (P = 0.69). The same phenomenon can also be observed in the soaked cotton pad experiment (Fig. 8b). Group A showed a statistically significant decrease (0.051/mm, P = 0.01) in the scattering coefficient after the removal of the soaked cotton pad, while there was no significant difference observed in Group B (P = 0.14).

The phenomenon of decreased scattering has also been observed in other studies. From Tuchin and his team’s observation on skin surfaces applied with cosmetic gel, they noticed a decrease in the scattering coefficient, while absorption coefficient slightly increased^40^. According to their Mie scattering simulation, they mentioned that scattering strongly depends on both the particle size and relative refractive index^40^. Based on the results discussed earlier, we inferred that the strong sensation of tightness in Group A is due to cell swelling, which causes photons to refract less in the tissue, thereby reducing the scattering coefficient. As for Group B, the moisture is contained at shallower layers, the cells didn’t swell up, so there’s no significant decrease in the scattering coefficient. In summary, differences in scattering coefficient were observed through the grouping of subjects into Groups A and B, which suggests a relationship between scattering coefficient and the sensation of tightness. Scattering coefficient may be useful in evaluating cellular swelling. In this study, we observed a decrease in the 980 nm scattering coefficient during skin hydration, and we speculate that the 970 nm scattering coefficient is also affected. To provide a more comprehensive understanding, we are planning subsequent experiments that will extend our analysis across a wider range of wavelengths. This will enable us to better interpret the changes in scattering profiles in relation to cell hydration and swelling, offering a more holistic view of the effects of water content on skin optical properties. In addition, statistically significant differences were observed in both TEWL (Fig. 7b) and reduced scattering coefficient (Fig. 8a) between before and after facial mask treatment in Group A, while there were no such differences in Group B. The performance of TEWL and reduced scattering coefficient shows similarities, and there might be a correlation between the two that requires further experimentation to confirm. However, it is important to note that immediate measurements after hydrating treatments can yield elevated TEWL values, which may not truly reflect the skin's barrier function but rather the immediate effects of the treatment. Our study results suggest that TEWL may not be suitable for analyzing short-term changes following hydrating treatments. Furthermore, while TEWL is a commonly used indicators for skin barrier function, it may not serve as the best reference methods due to its limitation in z-axis resolution.

## Methods

### DRS system configuration

**Figure 9:**
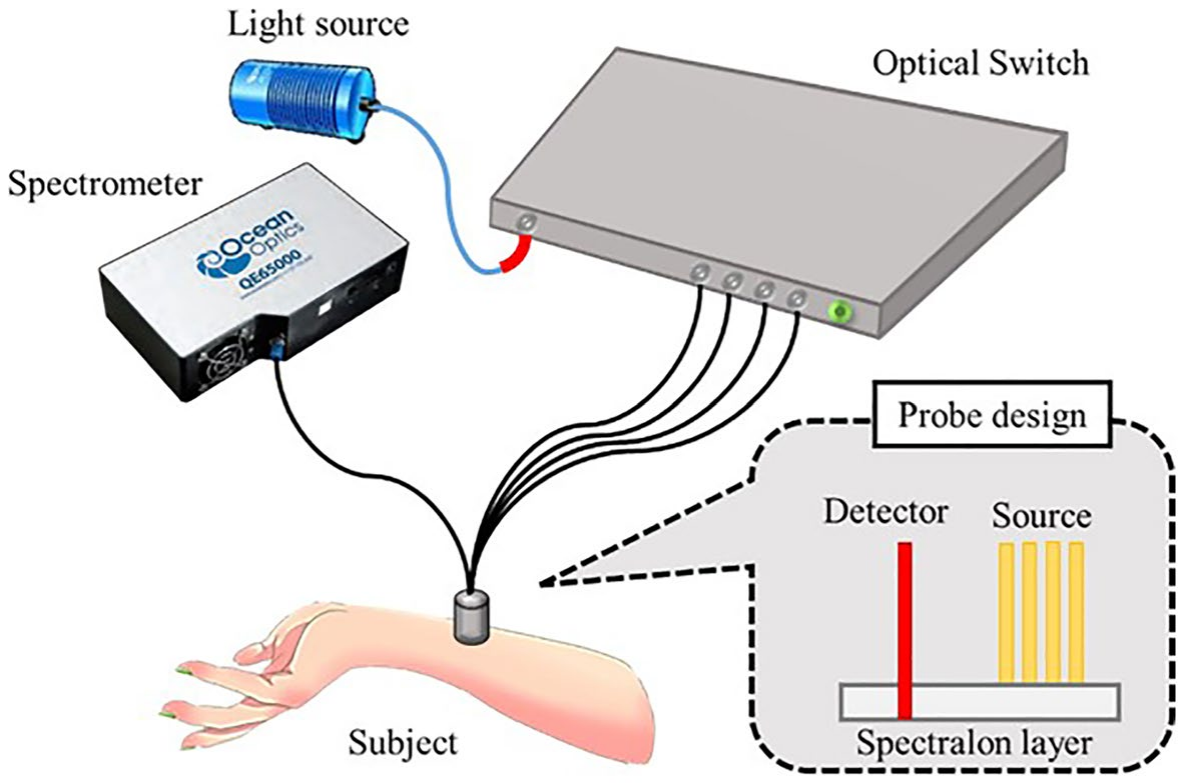
Configuration of the diffuse reflectance spectroscopy system and the diffusing probe used in this study.

The spatially resolved DRS system used in the present study is illustrated in Fig. 9. Diffuse reflectance from the tissues was collected by using a diffusing probe. The optical properties of the skin were determined with a simplified photon diffusion equation, given that the diffusing probe was equipped with a high scattering Spectralon slab (Labsphere, NH, USA), which efficiently diffused the light source. Four source fibers were placed on the upper surface of the Spectralon, and the detection fiber penetrated through the 1 mm Spectralon layer to be flush with the lower surface of the Spectralon. The detector fiber was connected to a spectrometer (QE65000, Ocean Optics, FL, USA). Four other fibers were connected to the light source through an optical switch (Piezosystem Jena, Germany). All optical fibers employed in the probe were multimode fibers with a 440 µm core diameter and 0.22 numerical aperture. Considering the fiber diameters and fabrication limitations, the source-to-detector separations were set to 1.44, 1.92, 2.40, and 2.88 mm for measurement of the skin hydration of subjects. Our light source was a broadband Tungsten Halogen light source (HL2000, Ocean Optics, FL), which provided a continuous spectrum of 400–1000 nm. The spectrometer and optical switch were connected to a laptop and controlled using MATLAB software (MathWorks, Natick, MA, USA).

### Theoretical models and skin optical properties recovery

In previous publications, the photon propagation model for the diffusing probe has been described in detail^29,37,55,56^. Here we summarize the key steps of the model derivation. In a two-layer turbid medium system, the diffusion equation is:

1 $$ \left[ {\frac{1}{{c_{i} }}\frac{\partial }{\partial t} + \mu_{ai} - \nabla \left[ {D_{i} \left( r \right)\nabla } \right]} \right]\Phi_{i} \left( {r,t} \right) = S_{i} \left( {r,t} \right) $$

where *c* is the speed of light in the medium, *D* = 1/3(*µ*_*a*_ + *µ*_*s*_*'*) and Φ are the diffusion constant and the fluence rate, respectively. *S* is the source term, and *i* = 1, 2 is the number of the layer. The light source is expressed as *S*_1_ = *δ*(*x*,*y*,*z*-*z*_0_) and *S*_2_ = 0, where *z*_0_ = 1/(*µ*_*a*_ + *µ*_*s*_*'*) is the location of the point source ^57^. The detector in the modified two-layer geometry is located at the boundary of the first layer and the second layer. In the Fourier domain, the fluence rate at the detector should be expressed as:

2 $$ \varphi_{2} (z,s) = \frac{{\sinh [\alpha_{1} (z_{b} + z_{0} )]}}{{D_{1} \alpha_{1} \cosh [\alpha_{1} (l + z_{b} )] + D_{2} \alpha_{2} \sinh [\alpha_{1} (l + z_{b} )]}} $$

where $\varphi_{2} (z,s) = \int_{ - \infty }^{\infty } {\int_{ - \infty }^{\infty } {\Phi_{2} (x,y,z)\exp [i(s_{1} x + s_{2} y)]dxdy} }$, $\alpha_{2}^{2} = (D_{2} s^{2} + \mu_{a2} + j\omega /c)/D_{2}$, *l* is the thickness of the first layer, and $s^{2} = s_{1}^{2} + s_{2}^{2}$. The fluence rate at the detector could be determined by inverse Fourier transform. The spatially resolved reflectance could be calculated as the integral of the radiance *L*_2_ at the boundary, where $L_{2} = \Phi_{2} + 3D_{2} (\partial \Phi_{2} /\partial z)\cos \theta$, over the backward hemisphere, as follows:

3 $$ R\left( \rho \right) = \mathop \smallint \nolimits_{2\pi }^{{}} \left[ {1 - R_{fres} \left( \theta \right)} \right]\cos \theta \left( {L_{2} /4\pi } \right)d{\Omega } $$

here, $\rho = \sqrt {x^{2} + y^{2} }$ and *R*_*fres*_(*θ*) is the Fresnel reflection coefficient for a photon with an incident angle of *θ* relative to the normal to the boundary^35^. To remove the instrument response, each set of skin reflectance spectrum was first calibrated by the reflectance spectrum measured from a silicone phantom with known optical properties. With the use of the two-layer photon diffusion model and a least-squares curve-fitting algorithm (“lsqcurvefit” nonlinear curve fitting function in MATLAB), an absorption spectrum and a reduced scattering spectrum of the turbid sample could be derived.

### Two skin hydration treatments and cutaneous sensation questionnaires

**Figure 10:**
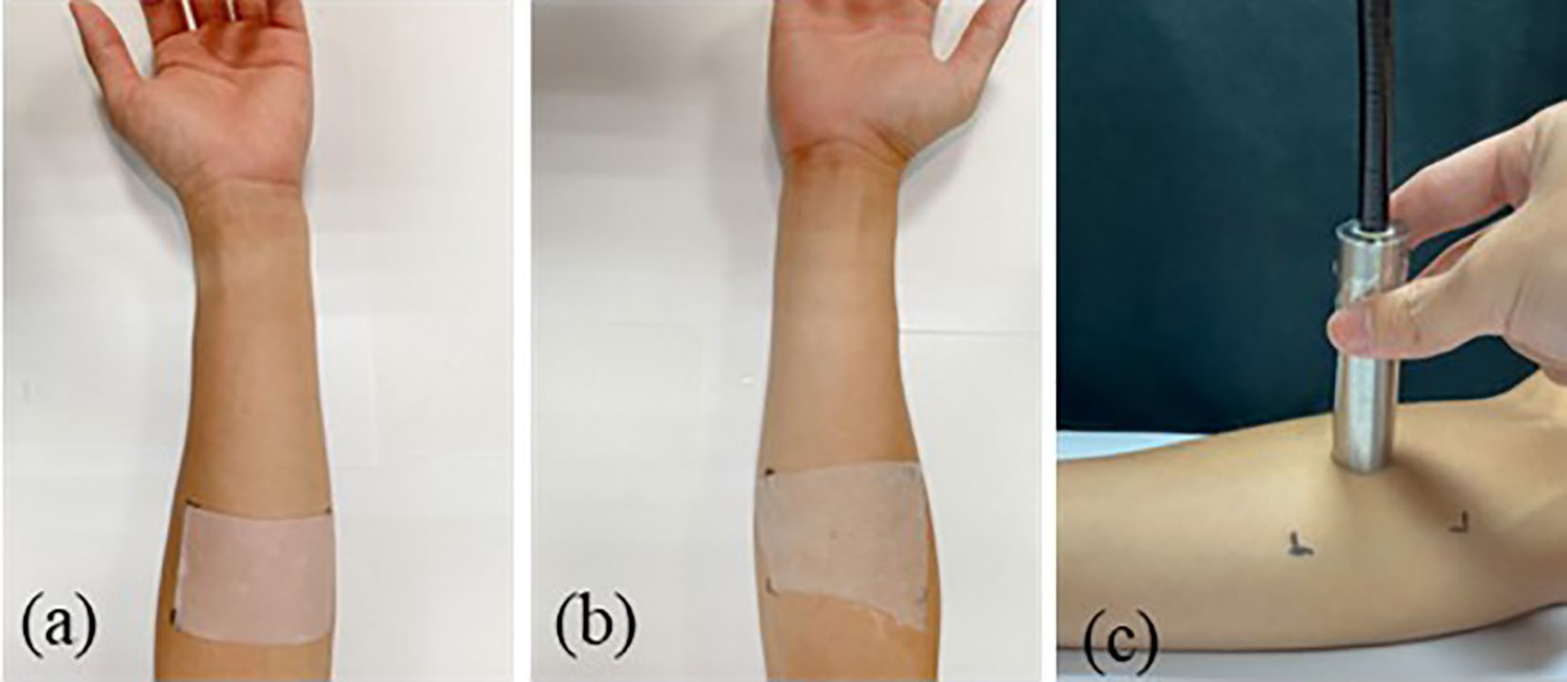
(**a**) Subject’s left ventral forearm with a soaked cotton pad. (**b**) Subject’s right ventral forearm with a commercially available facial mask. (**c**) Picture of skin measurement using the fiber optical probe.

This in-vivo skin hydration study included 10 healthy subjects. The volunteers, who gave informed written consent, consisted of 5 females and 5 males aged between 20 and 30 years old. The protocol was approved by the National Cheng Kung University Institutional Review Board, Taiwan (No. NCKU HREC-E-112-400-2). Written informed consents were obtained from all the subjects in the study. The study was conducted in accordance with the latest revision of the Declaration of Helsinki. They were non-smokers and did not have any known or visible skin diseases. The subjects were asked not to use any skin products on the day of the measurements. During the measurements, the indoor relative humidity was kept at 50 ± 4% and the temperature was kept at 25 ± 1 °C. In the beginning of the experiments, the subjects’ forearms were gently cleaned with water and then rested for one hour. An area of 5 × 7.7 cm^2^ was marked on the ventral forearms of each subject and defined as the skin measurement sites. Cotton pads (KNH Enterprise CO., Ltd., Taiwan) were prepared by soaking them in DI water. Subsequently, three baseline measurements were performed on the measurement sites to ensure the stability of our system as well as the skin condition. As soon as the baseline measurements were finished, the left and right volar forearms of each subject were treated with 5 × 7.7 cm^2^ soaked cotton pad and commercially available moisturizing facial mask (DR. JOU Co., Ltd., Taiwan) for 20 min, respectively, as shown in Fig. 10a and b The primary components of this facial mask include purified water, allantoin, PCA-Na moisturizing factor, vitamin B5, large and small molecule hyaluronic acid, among others. After the treatments, the treated areas were wiped with tissue paper to remove any remaining water on the skin surface. Measurements were further performed at the time points of 0, 5, 10, 20, 40, and 60 min after removing the facial mask or cotton pad. The measurement was performed by gently placing the optical probe at the center of the skin measurement site as shown in Fig. 10c. Each optical measurement contained 10 readings, and the average value was taken as the measurement value.

In addition, in this study, we used GPSkin Barrier® (GPOWER Inc., Korea) to perform TEWL and skin relative electrical conductivity measurements^58–60^. GPSkin Barrier® uses two electronic sensors at the edge of the probe to measure the capacitance of the skin, and then convert it into a reference value of skin hydration level, which falls between 50 and 100. For TEWL measurement, the probe contains a pseudo-closed chamber system with temperature and humidity sensors^61^. The TEWL and relative electrical conductivity values determined by GPSkin Barrier® have been proven to correlate well with skin water content reference values and epidermal permeability barrier function on the cheek, the dorsal hand, and the forearm in a past study with 200 normal volunteers^62^.

Furthermore, it has been reported in related skin hydration studies that research groups used questionnaire ratings to conduct qualitative analyses on skin sensation^15^. Given the current lack of instrumentation specifically designed to quantify skin sensations, we opted for a subjective approach in this study, utilizing two different hydration treatments to help the recruited subjects compare the differences in sensations. Thus, in this study, questionnaires to evaluate cutaneous sensation were obtained at 0, 5, 10, 20, 40, and 60 min, after removal of facial mask and soaked cotton pad. A 6-grade score for cutaneous sensation, including redness, whiteness, stickiness, elasticity, and tightness, was self-evaluated by each participant.

### Statistical methods

A portion of the data in this study is presented in the form of mean ± standard error of the mean (s.e.m.). The mean represents the average value of the data, while the s.e.m. provides an estimate of the variability or precision of the mean. To evaluate the variations in absorption coefficient, scattering coefficient, relative electrical conductivity, and transepidermal water loss (TEWL) following the skin hydration treatments, we conducted statistical analysis using Student's t-test. Unless otherwise mentioned, in this study the P values were determined using a two-tailed and paired Student's t-test for the 10 participants included in the study. The statistical significance was defined as P < 0.05. Box-and-whisker plots were generated to describe the statistics summary using OriginPro2021 software (OriginLab, USA) and will be shown in Discussion. B. In the plots, the length of the box represents the distance between the 25th and 75th percentiles, the symbol in the box represents the group mean, the horizontal line in the box represents the group median, the whiskers issuing from the box extend to the group minimum and maximum values, and the outliers are shown in asterisks.

## Data Availability

The datasets used and/or analysed during the current study are available from the corresponding author on reasonable request.
